# Combination immunotherapy with anti-PD-L1 antibody and depletion of regulatory T cells during acute viral infections results in improved virus control but lethal immunopathology

**DOI:** 10.1371/journal.ppat.1008340

**Published:** 2020-03-30

**Authors:** Paul David, Malgorzata Drabczyk-Pluta, Eva Pastille, Torben Knuschke, Tanja Werner, Nadine Honke, Dominik A. Megger, Ilseyar Akhmetzyanova, Namir Shaabani, Annette Eyking-Singer, Elke Cario, Olivia Kershaw, Achim D. Gruber, Matthias Tenbusch, Kirsten K. Dietze, Mirko Trilling, Jia Liu, Dirk Schadendorf, Hendrik Streeck, Karl S. Lang, Youhua Xie, Lisa Zimmer, Barbara Sitek, Annette Paschen, Astrid M. Westendorf, Ulf Dittmer, Gennadiy Zelinskyy

**Affiliations:** 1 Institute for Virology, University Hospital Essen, University of Duisburg-Essen, Essen, Germany; 2 Institute of Medical Microbiology, University Hospital Essen, University of Duisburg-Essen, Essen, Germany; 3 Department of Rheumatology, Hiller Research Center Rheumatology, University Hospital Düsseldorf, Germany; 4 Medizinisches Proteom-Center, Ruhr-Universität Bochum, Bochum, Germany; 5 Department of Pathology, Albert Einstein College of Medicine, New York, New York, United States of America; 6 Institute of Immunology, University Hospital Essen, University of Duisburg-Essen, Essen, Germany; 7 Department of Gastroenterology and Hepatology, University Hospital Essen, University of Duisburg-Essen, Essen, Germany; 8 Department of Veterinary Medicine, Institute of Veterinary Pathology, Free University Berlin, Berlin, Germany; 9 Institute of Clinical and Molecular Virology, University Hospital Erlangen, Friedrich-Alexander University Erlangen-Nürnberg, Erlangen, Germany; 10 Department of Infectious Diseases, Union Hospital of Tonji Medical College, Huazhong University of Science and Technology, Wuhan, China; 11 Department of Dermatology, Comprehensive Cancer Center, University Hospital Essen, Essen, Germany; 12 Institute for HIV Research, University Hospital Essen, University Duisburg-Essen, Essen, Germany; 13 Key Lab of Molecular Virology, Shanghai Medical College, Fudan University, Shanghai, China; Vaccine Research Center, UNITED STATES

## Abstract

Combination immunotherapy (CIT) is currently applied as a treatment for different cancers and is proposed as a cure strategy for chronic viral infections. Whether such therapies are efficient during an acute infection remains elusive. To address this, inhibitory receptors were blocked and regulatory T cells depleted in acutely Friend retrovirus-infected mice. CIT resulted in a dramatic expansion of cytotoxic CD4+ and CD8+ T cells and a subsequent reduction in viral loads. Despite limited viral replication, mice developed fatal immunopathology after CIT. The pathology was most severe in the gastrointestinal tract and was mediated by granzyme B producing CD4+ and CD8+ T cells. A similar post-CIT pathology during acute Influenza virus infection of mice was observed, which could be prevented by vaccination. Melanoma patients who developed immune-related adverse events under immune checkpoint CIT also presented with expanded granzyme-expressing CD4+ and CD8+ T cell populations. Our data suggest that acute infections may induce immunopathology in patients treated with CIT, and that effective measures for infection prevention should be applied.

## Author summary

Combination immunotherapy (CIT) directed against checkpoint mechanisms has been approved for the therapy of cancers and is proposed for the treatment of chronic infections. In cancer therapy patients often develop severe immunopathology under CIT. Here we show that acute viral infections (Friend retrovirus and Influenza virus) posed a significant threat during CIT in mice. The strong activation of cytotoxic T cells after an acute viral infection seemed to lack couterregulation during CIT, which resulted in lethal immunopathology. In case of an Influenza virus infection this could be prevented by vaccination prior to CIT. The expansion of CD4+ and CD8+ T cells expressing cytotoxic molecules were also observed in melanoma patients treated with two checkpoint blockers. Thus, these patients might also be at risk of developing severe immunopathology during an otherwise harmless acute viral infection. This should to be taken into account when cancer patients are undergoing CIT.

## Introduction

Cytotoxic CD8^+^ T cells are crucial for the control of tumor cells and acute viral infections. However, during chronic viral infections, like HIV or HCV, these cells become dysfunctional or “exhausted” [1]. The studies of Dr. Ahmed`s group [2] demonstrated that signaling from the programmed death receptor (PD-1) expressed on LCMV specific CD8^+^ T cells is a central mechanism regulating the development of T cell dysfunction during chronic viral infections. Blocking the PD-1/PD-L1 interaction and/or other inhibitory receptors, like Tim-3, therefore results in improved control of chronic infections. These studies and previous work in tumor mouse models [3,4] paved the way to develop new immunotherapies against certain human cancers, as T cell dysfunction is also a hallmark of many malignant diseases [5]. The clinical success of these immunotherapies has been outstanding for some cancer entities treatment of melanoma, non-small cell lung cancer, renal cell carcinoma, colorectal cancer, hepatocellular carcinoma, Hodgkin lymphoma and others [6].

Recently, it became evident that activated virus-specific CD8+ T cells also express PD-1 and other inhibitory receptors in the acute phase of viral infections [7–9]. During this phase, T cells are not dysfunctional, but inhibitory receptors seem to regulate the overall magnitude of the T cell response. Thus, the treatment of mice with anti-PD-L1 or anti-PD-1 antibodies enhanced the magnitude of antiviral immune responses during acute infections and improved virus control [9,10]. Experiments with PD-1 or PD-L1 KO mice also demonstrated enhanced antiviral CD8+ T cell response against acute viral infections [11–13]. Thus, inhibitory receptors have important functions in regulating antiviral cytotoxic T cell responses during acute and chronic viral infections.

Previously, others and we showed that also regulatory T cells (Tregs) contribute to the exhaustion of the T cell response during chronic infections [14]. Tregs are a population of forkhead box P3 (Foxp3) expressed lymphocytes which suppress effector CD4+ and CD8+ T cells to prevent the development of autoimmunity, but they also suppress T cell responses during infectious diseases [15] and different cancers [16]. The suppression of antiviral T cell responses by Tregs was first described in studies using the Friend retrovirus (FV) infection of mice [17]. FV is a haematotropic retroviral complex that induces erythroleukemia in susceptible mice. In contrast, resistant strains, like C57BL/6 mice that were used in the current study, develop potent immune responses during acute infection that allow protection from leukemia, but they establish a life-long chronic infection when challenged with a high dose of the virus [18,19]. During chronic FV infection, transient depletion of Tregs reactivated exhausted CD8^+^ T cells and thereby significantly reduced chronic viral set points [20]. Treg-mediated suppression of effector T cell responses was also observed in chronic infections of humans, including HIV and HCV [21]. Similar results were obtained during acute FV infection in which depletion of Tregs resulted in enhanced effector T cell functions and decreased viral loads [22,23]. However, in each experimental treatment alone, the blocking of the PD-1/PD-L1 pathway or Treg ablation did not result in complete viral clearance during acute FV infection, and thus chronic infection could still develop. Therefore, combining the two therapies might be a promising approach to further augment the effects of immune checkpoint blockers in an acute retroviral infection.

Indeed, recent studies showed that the effect of immunotherapy with anti-PD-L1 can be potentiated by combining it with other treatments targeting another mechanism of immunoregulation [24]. For chronic viral infections this has been demonstrated *in vivo* using two different mouse models. The effect of anti-PD-L1 treatment of chronic FV infection was significantly enhanced after additional Treg depletion [25]. The combination treatment resulted in an expansion of cytotoxic CD8^+^ T cells and a reduction of FV loads in infected organs. Similar results were obtained in persistent LCMV infection [26], and in LP-BM5 murine leukemia retrovirus induced acquired immunodeficiency syndrome termed murine AIDS (MAIDS)[27]. In mice, which received combined treatment with anti-PD-L1 antibodies and depletion of Tregs, the reduction in chronic viral loads was significantly greater than with either treatment alone. Thus, combination therapies targeting inhibitory receptors and Tregs at the same time have been suggested for the therapy of infectious diseases and cancers in many recent publications. In fact, the very successful combination therapy in malignant melanoma patients using anti-PD-L1 plus anti-CTLA-4 antibodies was reported to block inhibitory receptors as well as Treg functions [28,29].

The initial idea of the current study was to combine the therapeutic depletion of Tregs with the blockade of PD-L1 and Tim-3 as treatment during acute FV infection, with the ultimate goal to prevent the establishment of viral chronicity. Our data indeed shows a massive expansion of cytotoxic T cells followed by a significant reduction or even complete elimination of FV. However, all mice receiving combination treatment developed fatal systemic immunopathology shortly after the therapy. To verify these findings with a virus that has more relevance for human infections, we repeated the experiment with Influenza A (IA) virus. Again all animals died of immunopathology after immunotherapy, but could be rescued by IA vaccination. The current study supplies important evidence that an otherwise harmless acute viral infection can result in severe pathology if immune checkpoints are manipulated. This needs to be taken into account, when cancer patients are considered to be treated with combination immunotherapy.

## Results

### Combination therapy activates cytotoxic T cells and reduces acute viral loads

**Fig 1:**
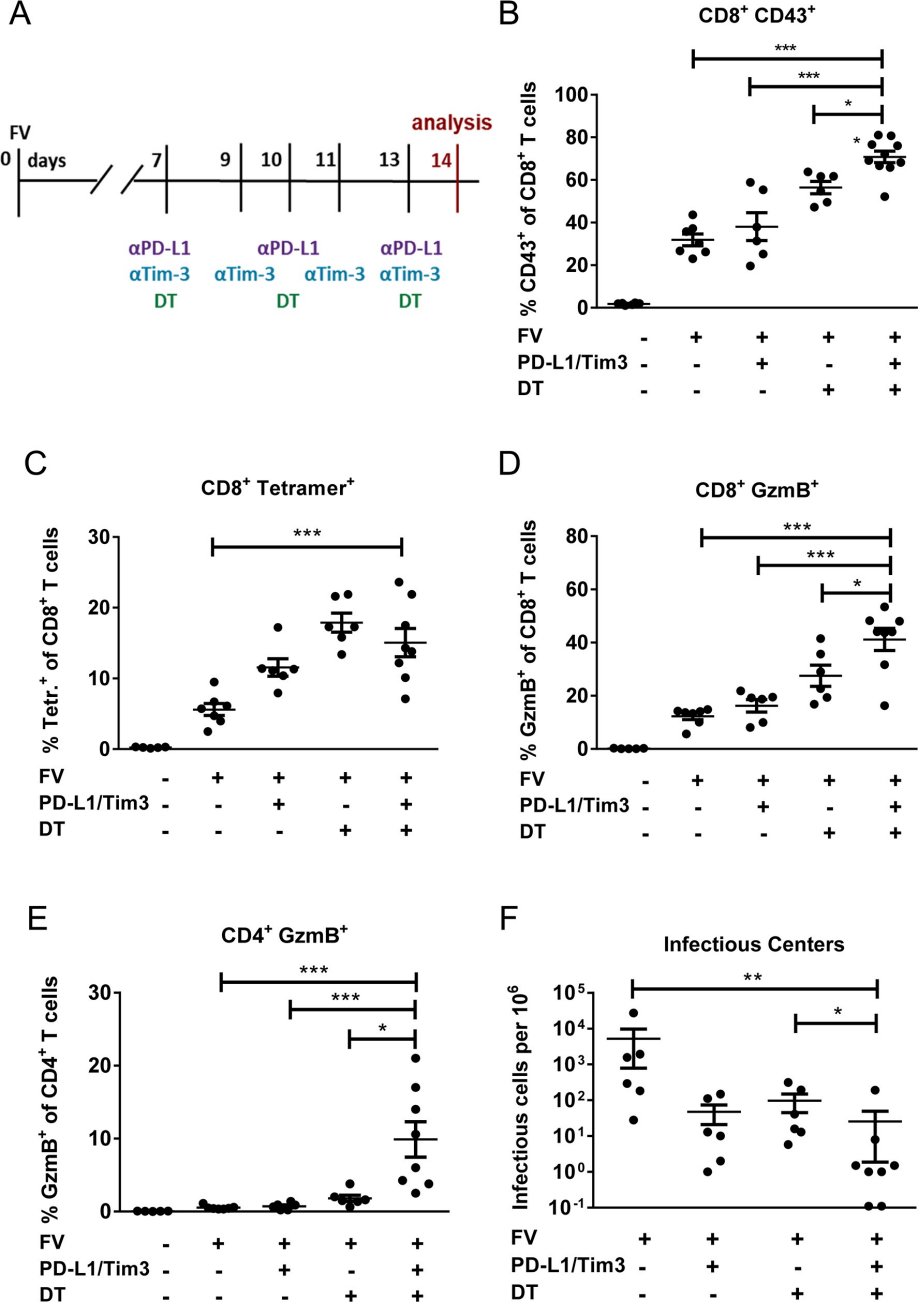
Reduced levels of FV and enhanced number of CD8+ and CD4+ T cells producing GzmB after Treg depletion and/or inhibitory pathway blockage in acutely infected mice. DEREG mice were infected with FV and treated with DT and/or blocking antibodies against PD-L1 and TIM-3 as indicated (**A**). Flow cytometry was used for the determination of spleen CD8+ T cells which are expressing CD43 (**B**), positive for MHC class I H2-Db tetramers specific for FV GagL peptide (Tetr+) (**C**), and producing the GzmB (**D**), and the percentages of CD4+ T cells producing the GzmB (**E**). Spleens of chronically FV-infected mice from the different treatment groups were analyzed for viral loads by infectious center assays one day after termination of treatment (**F**). Each dot represents an individual mouse. Data were pooled from 3 to 5 independent experiments with similar results. Statistically significant differences are indicated by asterisks (* < 0.05; ** < 0.005; *** < 0.0005; one-way ANOVA with a Tukey post-test).

Since combination immunotherapy targeting Tregs and inhibitory receptors previously showed high efficacy for the treatment of chronic FV [25] and LCMV [26] infection, we investigated if such treatment during acute FV infection may result in complete elimination of the virus and thus prevents the establishment of viral chronicity. For the current experiments the DEREG strain of mice with C57BL/6 background was used that express diphtheria toxin (DT) receptor under the control of the Foxp3 promoter [30]. Injection of DT resulted in the specific elimination of Tregs, which was combined with an injection of blocking antibodies against PD-L1 and Tim-3. The treatment was performed during the second week of FV infection (day 7 to 13) and the spleens were analyzed directly after treatment on day 14 after infection (Fig 1A). Mice were treated with either treatment alone (Treg depletion or inhibitory receptor block with the two antibodies) or a combination of the two.

Cytotoxic effector CD8+ T cells are the main immune cell population responsible for the control of FV during acute infection [31] and the total population of effector CD8+ T cells responding to FV infection can be determined by the expression of the activation-associated glycoform of CD43 [31,32]. The highest frequencies of CD8+CD43+ T cells were observed in the group of mice receiving combination therapy, which were significantly higher compared to individual treatments (Fig 1B, S1A Fig). Interestingly, the frequencies of CD8+ T cells specific for the immunodominant FV epitope D^b^-GagL [33] (Tetr+) were enhanced after the antibody or DT treatment alone, but the combination of the two did not further increase percentages of virus-specific CD8+ T cells (Fig 1C, S1B Fig). CD8+ T cells contain granula with cytotoxic molecules, like the serine protease granzyme B (GzmB). Percentages of CD8+ GzmB+ T cells were significantly enhanced in mice receiving combination immunotherapy in comparison to any monotherapy (Fig 1D, S1C Fig). Moreover, we also analyzed whether immunotherapy induces cytotoxic CD4+ T cells. Previous studies reported that during acute FV infection virus-specific CD4+ T cells develop a Th1 or TfH-like phenotype with no signs of cytotoxic activity [34, 35]. Accordingly, GzmB expression in CD4+ T cells was not detected in FV-infected mice or animals receiving either Treg or inhibitory receptor therapy, but approximately 10% of the CD4+ T cells from mice on combination therapy started to produce GzmB (Fig 1E, S1D Fig). The augmented cytotoxic T cell responses after combination treatment were expected to result in reduced viral loads in these mice. Indeed, it was associated with a dramatic reduction of FV loads (Fig 1F) and in some mice of this group (about 25%) infectious virus was no longer detectable with an infectious center assay. These data suggested that combination immunotherapy may be a promising approach for the treatment of acute virus infections, which may prevent the establishment of viral chronicity.

### Combination therapy induces immunopathology during acute FV infection

**Fig 2:**
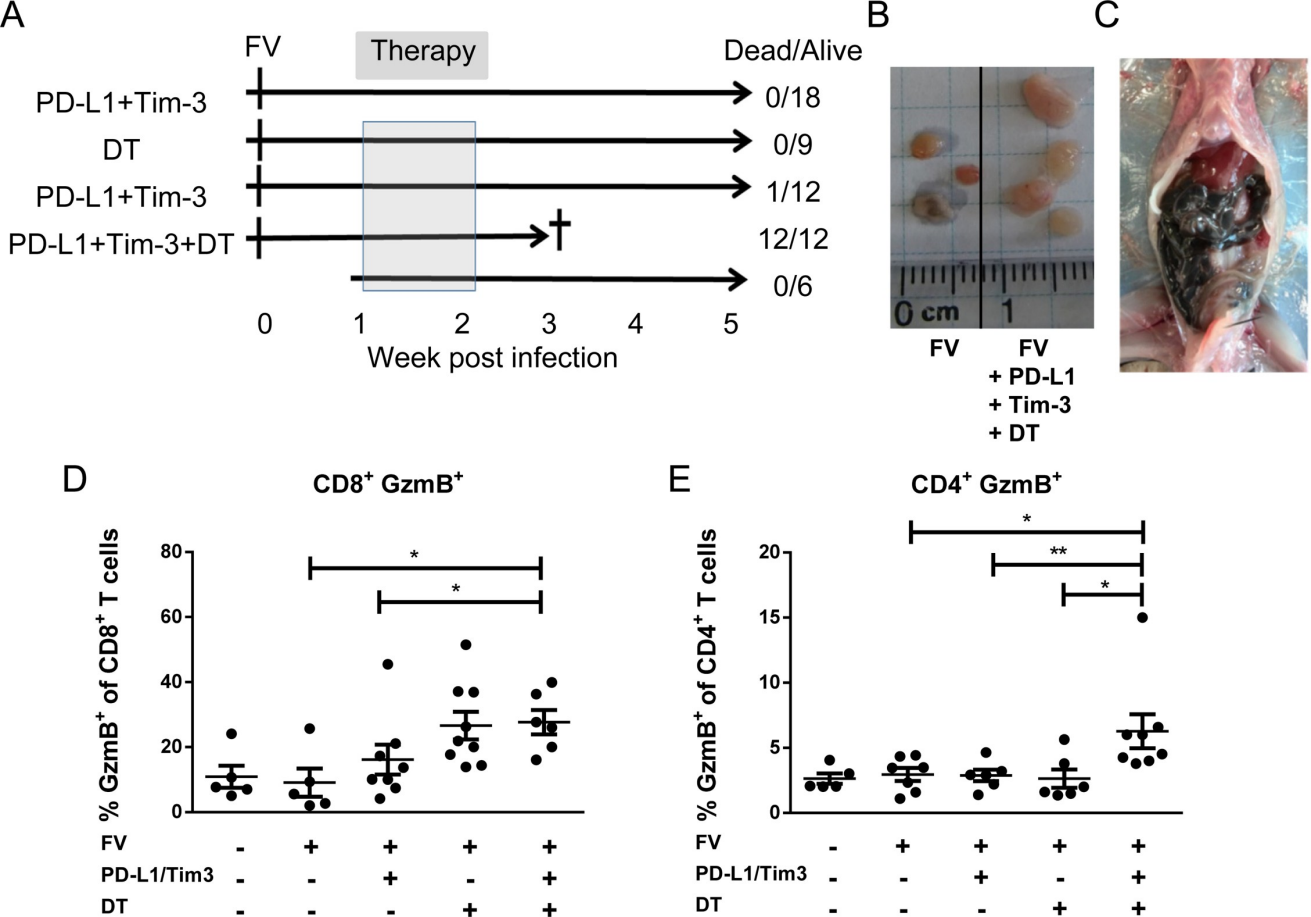
The death of FV infected mice within one week after Treg depletion and blocking of inhibitory pathways. DEREG mice were infected with FV and were treated with DT and/or blocking antibodies against PD-L1 and TIM-3 as indicated during the second week of infection (**A**). Lymphadenopathy (**B**) and pathology of the intestine (**C**) in mice after Treg depletion and treatment with anti-PD-L1 and anti-TIM-3. The percentages of CD8+GzmB+ (**D**) or CD4+GzmB+ (**E**) T cells isolated from the intestine of mice infected with FV and treated with DT and/or blocking antibodies against PD-L1 and TIM-3. Data were pooled from 3 to 5 independent experiments with similar results. Statistically significant differences are indicated by asterisks (* < 0.05; ** < 0.005; one-way ANOVA with a Tukey post-test).

In order to test if combination treatment can indeed prevent viral chronicity the treatment protocol during the acute phase of FV infection was repeated, but the analysis of the mice was scheduled for week 6 post infection (chronic phase). Surprisingly, all mice (12 out of 12) that received the combination therapy developed lethal pathology and were sacrificed between day 5 and 7 post treatment (Fig 2A). Mice from the other groups survived without any symptoms of morbidity, except one mouse (1 out of 12) in the Treg depletion group developed pathology. Also control mice from a group of non-infected animals receiving combination therapy survived, indicating that only the combination of acute FV infection with two checkpoint blocking therapies was fatal for the mice. We therefore investigated why mice in the combination therapy arm died despite low viral load and indication of a cure. In order to address this question, pathological and immunological studies were performed on day 4 after treatment (18 days after infection). Close observation of mice showed reduced motility, a reduced consumption of food, and apathy in the group of FV-infected mice receiving the combination treatment. The animals from all other groups did not show any abnormal behavior. An autopsy of mice after combination therapy revealed systemic lymphadenopathy (Fig 2B). The pathological analysis showed hepatitis, pancreatitis, and gastritis in mice after FV infection and combination treatment (S2 Fig). Moreover, the small intestine of these mice was swollen and filled with melena (Fig 1C). The intestines of mice from the different groups were isolated and characterized by immunohistochemical staining for the infiltration of T lymphocytes. The intestinal crypts of all analyzed mice contained CD4+ and CD8+ T cells (S3 Fig). The numbers of CD4+ T cells in these crypts were much higher than those of CD8+ T cells, but no obvious differences between the therapy groups were found. In the following experiments T cells from the intestinal lamina propria were isolated and characterized for GzmB expression. The combination treatment led to a significantly higher frequency of intestinal CD8+ T cells producing the cytotoxic molecule GzmB in FV-infected mice or mice receiving only blocking antibodies against inhibitory receptors compared to non-treated FV-infected animals (Fig 2D). Similarly, high frequencies of CD8+ GzmB+ T cells were found in only Treg depleted mice. In contrast, an enhanced frequency of GzmB producing CD4+ T cells in the lamina propria was only found in FV-infected mice after combination therapy (Fig 2E). In all other groups of animals, the numbers of CD4+ T cells producing GzmB were unchanged after treatment.

**Fig 3:**
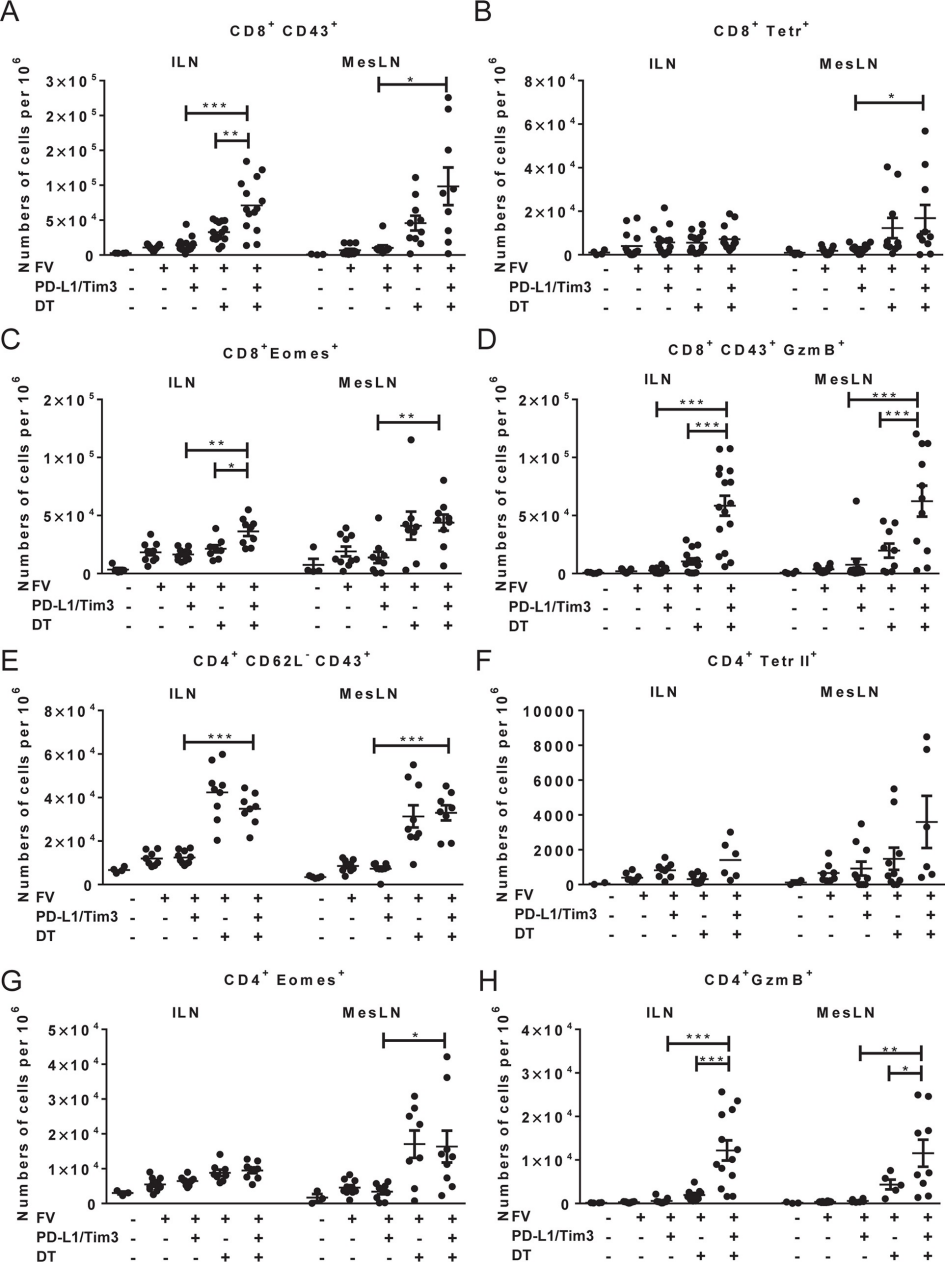
CD8+ and CD4+ T cell activity after combination immunotherapy. Mice were infected with FV and were treated with DT and/or blocking antibodies against PD-L1 and TIM-3 as indicated (Fig 1A). 18 days after infection inguinal lymph nodes (ILN) and mesenteric lymph nodes (MesLN) were isolated and the flow cytometry analysis of CD8+ and CD4+ T cells was performed. Frequencies of CD8+ T cells expressing CD43 (**A**) or positive for MHC class I H2-Db tetramers specific for FV GagL peptide (Tetr+) (**B**), or Eomes (**C**), or GzmB (**D**), are presented. The frequencies of CD4+ T cells expressing CD62L- CD43+ phenotype (**E**), or positive for MHC class-II Ab FV-envelope specific tetramer (Tetr II+) (**F**), or expressing Eomes (**G**), or GzmB (**H**) were detected. Each dot represents an individual mouse. Data were pooled from 3 to 5 independent experiments with similar results. Statistically significant differences are indicated by asterisks (* < 0.05; ** < 0.005; *** < 0.0005; one-way ANOVA with a Tukey post-test).

The pathology of the intestine was therefore associated with activation of the immune system and systemic lymphatic hyperplasia (Fig 2B). In order to determine the systemic activation of T cells, inguinal lymph nodes (ILN) were isolated and characterized by flow cytometry (S4 Fig). Percentages of CD8+ and CD4+ T cells expressing the transcriptions factor of effector T cells T-bet, the marker of proliferation Ki67, and surface molecules associated with an effector T cells phenotype (CD44+, CD43+, CD11a+, CD62L-, CD127-, CD69+) were strongly enhanced after combination therapy compared to all other groups. The ILN are localized subcutaneously and drain neither FV-infected organs nor organs with immunopathology. Thus, the expansion of activated T cells in ILN reflects the systemic activation of T cells after combination therapy. In the following experiments T cell functions were analyzed in the ILN and in mesenteric lymph nodes (MesLN). MesLN drain the gut and the activity of T cells in these LN reflects the T cell response in the intestine. The population of effector CD8+CD43+ T cells was significantly expanded in the ILN and MesLN after combination treatment (Fig 3A). However, no dramatic difference between the groups was found for numbers of virus-specific CD8+Tetr+ T cells (Fig 3B). Eomesodemin (Eomes) is a transcription factor necessary for the differentiation of cytotoxic CD4+ T cells [36, 37] and cytotoxic CD8+ T cells [38]. The combination treatment resulted in the highest detectable numbers of CD8+ T cells expressing Eomes (Fig 3C) or the cytotoxic molecule GzmB in both LN (Fig 3D), indicating that the cytotoxic program in CD8+ T cells is efficiently stimulated by the combination therapy.

To study CD4+ T cells responses during acute FV infection, we used the CD43+CD62L- phenotype that efficiently characterizes effector CD4+ T cells [34]. The depletion of Tregs alone or in combination with antibody treatment led to an expansion of CD4+ T cells with this phenotype in LN (Fig 3E). However, similar to the virus-specific CD8+ T cell response no dramatic expansion of CD4+ Tetr II+ T cells specific for MHC class-II A^b^ restricted envelope epitope of FV was detected in any of the groups (Fig 3F). The analysis of cytotoxic CD4+ T cells (Eomes+ or GzmB+ cells) showed significantly enhanced numbers of GzmB expressing CD4+ cells after combination treatment in both LN compared to either treatment alone (Fig 3H), whereas the differences for Eomes expression were not so striking between the groups (Fig 3G).

**Fig 4:**
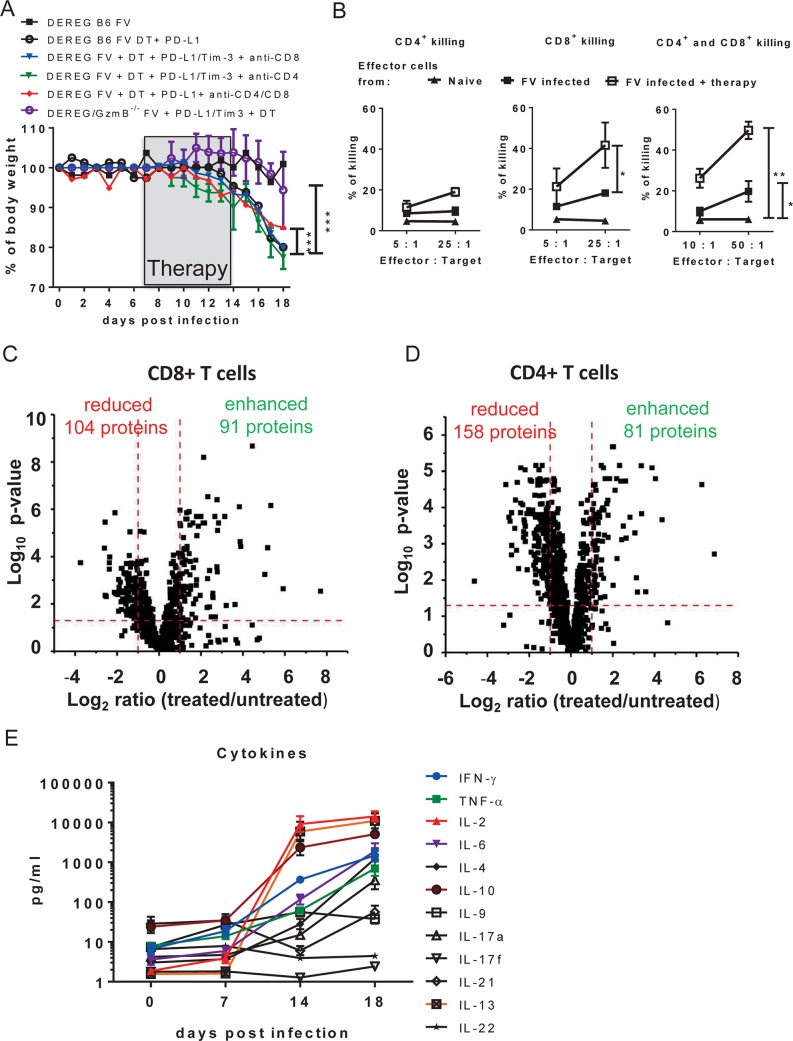
Functional properties of CD8+ and CD4+ T cells after combination immunotherapy. **A**. Weight of non-treated FV-infected mice, and mice infected and treated with DT and blocking antibodies against PD-L1 and TIM-3, and mice received additionally to combined treatment anti-CD4 or anti-CD8 or both anti-CD4 and anti-CD8 antibody, and DEREG-GzmB KO mice treated with DT and blocking antibodies against PD-L1 and TIM-3. For the depletion of CD4+ or CD8+ T cells mice were inoculated three times every other day starting on day 12 after FV infection. **B**. Redirect killing of P815 cells that were loaded with anti-CD3 antibodies and co-incubated with CD4+ T cells (left), CD8+ T cells (middle) and CD4+ plus CD8+ T cells (right) isolated from naïve mice, FV-infected mice and infected mice receiving combination treatment. Data were pooled from 3 independent experiments with similar results. Statistically significant differences are indicated by asterisks (* < 0.05; ** < 0.005; *** < 0.0005; non-parametric Mann-Whitney test). CD3+ CD8+ CD43+ T cells and CD3+CD4+ CD43+ CD62L- T cells were sorted from spleens of FV-infected DEREG mice and from FV-infected DEREG mice treated with DT and anti-PD-L1/anti-Tim-3 antibodies at 18 days post infection. After sorting cells were lysed for proteome analysis. Volcano plot showing the comparison of protein expression in CD3+ CD8+ CD43+ (**C**) and CD3+CD4+CD43+CD62L- (**D**) T cells isolated from non-treated and T cells with the same phenotype isolated from combination treated mice. Numbers of significantly reduced or enhanced proteins in the group of treated mice are shown in volcano plots. Benjamini–Hochberg corrected one-way ANOVA was used for the calculation of the p-values. **E.** FV infected mice from group received the combination treatment were bled at day 0, day 7, day 14 and day 18 after infection and the concentration of cytokines was analyzed. Data were pooled from 2 independent experiments with similar results.

To follow pathology the body weight of mice was compared between FV-infected mice, FV-infected mice receiving combination therapy, and FV-infected mice with combination therapy and T cell depletion. Acute FV infection alone did not result in any weight loss, whereas the combination treatment induced severe weight loss (Fig 4A), so that mice had to be euthanized. At 4 days post treatment the mean body weight of these mice was significantly lower than that of animals, which were additionally depleted for CD4+ and CD8+ T cells. Interestingly, the depletion of only CD4+ or only CD8+ T cells alone after combination treatment did not change the pathology in comparison to non-depleted mice. The results indicate that both T cell populations were indeed involved in immunopathology after the checkpoint blocking therapy during acute viral infection. The fact that T cell depleted mice still presented with a moderate weight loss after combination treatment might be explained by the incomplete depletion (spleen: 83% CD4+ and 94% CD8+ T cells; lymph nodes: 56% CD4+ and 74% CD8+ T cells) due to the strong activation of T cells. In order to show that GzmB dependent cytotoxicity played a key role in the development of immunopathology, DEREG mice were crossed with GzmB KO animals. DEREG-GzmB KO mice were then infected with FV and received combination therapy. Infected DEREG-GzmB KO mice did not lose any weight after the treatment, indicating that GzmB was critically involved in the pathology.

Since virus-specific CD4+ and CD8+ T cells that recognize immunodominant epitopes of FV did not significantly expand after combination therapy (Fig 1), it was possible that non-virus-specific cytotoxic CD4+ and CD8+ T cells might expand due to the therapy. To test this an *in vitro* killing assay for re-directed T cells was performed (Fig 4B) [39]. Mastocytoma P815 cells, which express Fc receptors were loaded with anti-CD3 antibodies and used as targets for killing. The re-directed killing of the P815 cells was induced after conjugation of cytotoxic T cells through the anti-CD3 antibody. The CD8+ and CD4+ T cells for this assay were separated from spleens of naïve mice, FV-infected mice, and from FV-infected mice receiving combination therapy. CD4+ and CD8+ T cells isolated from FV-infected, treated mice showed significantly higher cytotoxicity than cells isolated from FV-infected mice, with the highest killing seen in cultures containing a mixture of CD4+ and CD8+ T cells. Control cells, isolated from naïve mice did not show any cytotoxic function.

To further understand differences in the factors associated with this particular phenotype in animals receiving combination therapy, a global analysis of the CD4+ and CD8+ T cell proteome was performed by label-free quantification using liquid chromatography and tandem mass spectrometry (LC-MS/MS). At 18 days post infection CD3+CD8+CD43+ T cells and CD3+CD4+CD43+CD62L- T cells were sorted from the spleen of FV-infected DEREG mice and from infected DEREG mice treated with DT plus anti-PD-L1/Tim-3 antibodies. Cells were lysed and subjected to proteome analysis covering a total of 1889 proteins (S1 Table). From all detected proteins, the expression of 104 molecules was significantly enhanced and the expression of 91 proteins was reduced in CD8+ T cells isolated from mice receiving combination therapy (Fig 4C). Of these differently regulated proteins, 24 were associated with pathways of the immune system. In sorted CD4+ T cells from mice receiving combination therapy, the expression of 158 proteins was reduced and the expression of 81 proteins was enhanced in comparison to CD4+ T cells from non-treated FV-infected mice (Fig 4D). From those, 29 proteins were linked with the processes of the immune system. The following clustering analysis with the Gene Ontology enrichment tool (GO analysis) of differently expressed proteins showed that in both CD8+ and CD4+ T cells oxidation, metabolic processes, cell cycle, and apoptosis were influenced by therapy (S2 Table). Interestingly, the combination therapy led to enhanced expression of proteins that participated in endoplasmic reticulum stress. This cellular process is known to regulate the activation and the functionality of CD4+ [40] and CD8+ [41] T cells. Also, proteins which negatively affect apoptosis were upregulated after combination treatment. Thus, changes in the activation status and inhibition of apoptosis may be key cellular processes regulating the expansion of cytotoxic CD8+ and CD4+ effector T cells after combination therapy. The data suggest that the immunopathology in acutely infected mice after therapy was mediated by cytotoxic T cells. In order to test this hypothesis, both CD4+ and CD8+ T cells were depleted with monoclonal antibodies during the combination therapy in FV-infected mice.

Taken together, this data confirmed that GzmB-mediated cytotoxicity of both CD4+ and CD8+ T cells was critical for the development of immunopathology after combination therapy.

Immunopathology is often associated with a “cytokine storm” since activated effector T cells can produce large amounts of inflammatory cytokines. We, therefore, analyzed the levels of 12 different cytokines in the serum of FV-infected mice receiving combination therapy at different time points post infection to define the kinetics of the cytokine response. At 14 days post infection (directly after treatment) very high concentrations of IL-2, IL-13, IL-10, IFNγ, IL-6, and TNFα were observed (Fig 4E). During the following four days after treatment the concentration of these cytokines constantly increased demonstrating an on-going inflammatory response until the animals had to be euthanized at around 4 days post completion of therapy. The high levels of the anti-inflammatory cytokines IL-10 and IL-13 most likely reflected an insufficient counter-regulation to control the massive inflammatory response. This situation is characteristic for a “cytokine storm” [42] and reflects an unbalanced activation of the immune system. Summarizing the data from our immunological studies leads to the following conclusion: The combined immunotherapy blocking simultaneously the function of Tregs and inhibitory receptors during an acute viral infection induced the expansion of cytotoxic effector CD4+ and CD8+ T cells and the dysregulation of cytokine responses. The systemic immune activation resulted in immunopathology, which was most apparent in the intestines of FV-infected animals. Thus, combination immunotherapy targeting multiple immune checkpoint pathways may be harmful in an acute viral infection.

### The course of acute influenza virus infection in mice receiving immunotherapy

Given that combination therapies are already used for the treatment of cancers in humans [28], we next wanted to understand whether individuals receiving successful combination therapies are at risk for severe immunopathology during an acute viral infection. For that we used a clinically relevant human Influenza A (IV) virus mouse model.

**Fig 5:**
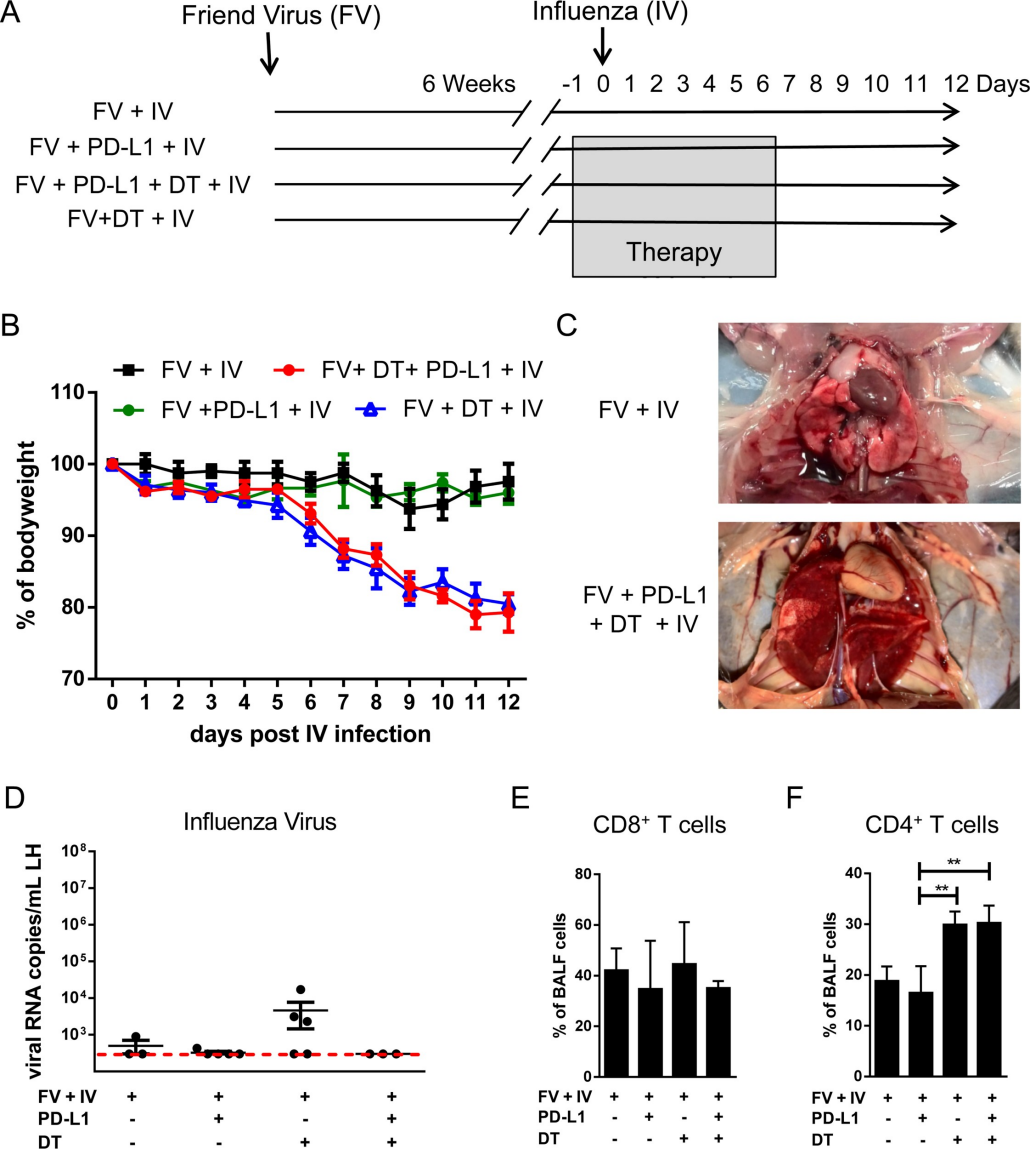
Lethality of chronic FV infected mice after Treg depletion and/or blocking of inhibitory pathways and infected with low doses of influenza virus. **A**. Chronically FV infected mice were treated with DT for Treg depletion and/or treated with anti-PD-L1 antibody. One day after starting the treatment mice were infected with IV. The weight of the analyzed group of mice (**B**) and the lungs of non-treated and combination treated mice (**C**). The numbers of RNA copies of IV in lung homogenate (**D**). The dashed line reflects the detection limit of the method. The percentage of CD4+ (**E**) and CD8+ (**F**) T cells in the BALF cells was calculated by flow cytometry. Each dot represents an individual mouse. Data were pooled from 2 to 3 independent experiments with similar results. Statistically significant differences are indicated by asterisks (** < 0.005; one-way ANOVA with a Tukey post-test).

The combination treatment with Treg depletion and blockade of PD-L1/Tim-3 was successfully used previously for the therapy of chronic FV infection [25]. Chronic FV infection does not induce any immunopathology in resistant mice and the combination therapy reduced chronic viral loads with no induction of clinical symptoms. In order to utilize this model, DEREG mice were infected with FV and rested for 60 days to establish viral chronicity. Chronically infected mice were treated with DT for the elimination of Tregs and/or with anti-PD-L1 antibodies (Fig 5A). In the current study we infected the mice intranasally with a low dose of influenza virus A during the period of immunotherapy. Non-treated mice and mice receiving only anti-PD-L1 antibodies controlled the low dose IV infection without obvious influenza symptoms including any detectible loss of weight (Fig 5B). In contrast, mice receiving the combination treatment or mice depleted for Tregs started to lose weight at day 6 after IV infection (Fig 5B). More than 20% loss of body weight was reached at day 12 after infection, which was the indication to stop the experiment. Treg depleted mice and mice receiving combination treatment were in poor condition, with liquid exudate in their lungs (Fig 5C), a typical symptom for advanced pneumonia. The analysis of lung homogenates for viral RNA showed very low numbers of IV RNA copies in some mice depleted for Tregs and no detectable virus in mice from the other groups (Fig 5D). The absence of viral RNA in lung tissue provided evidence that the observed pathology of the lung was not directly caused by IV infection, but suggested that it was mediated by immune cells. This was supported by the findings that significantly enhanced frequencies of CD4+ T cells were present in bronchoalveolar lavage fluids (BALF) from mice with lung pathology and weight loss (Fig 5F). In contrast, the percentage of CD8^+^ T cells in BALF was not significantly different between the groups of mice (Fig 5E). Thus, CD4+ T cells in BALF may be involved in the development of lung pathology in IV-infected mice that were Treg depleted or that received combination therapy.

**Fig 6:**
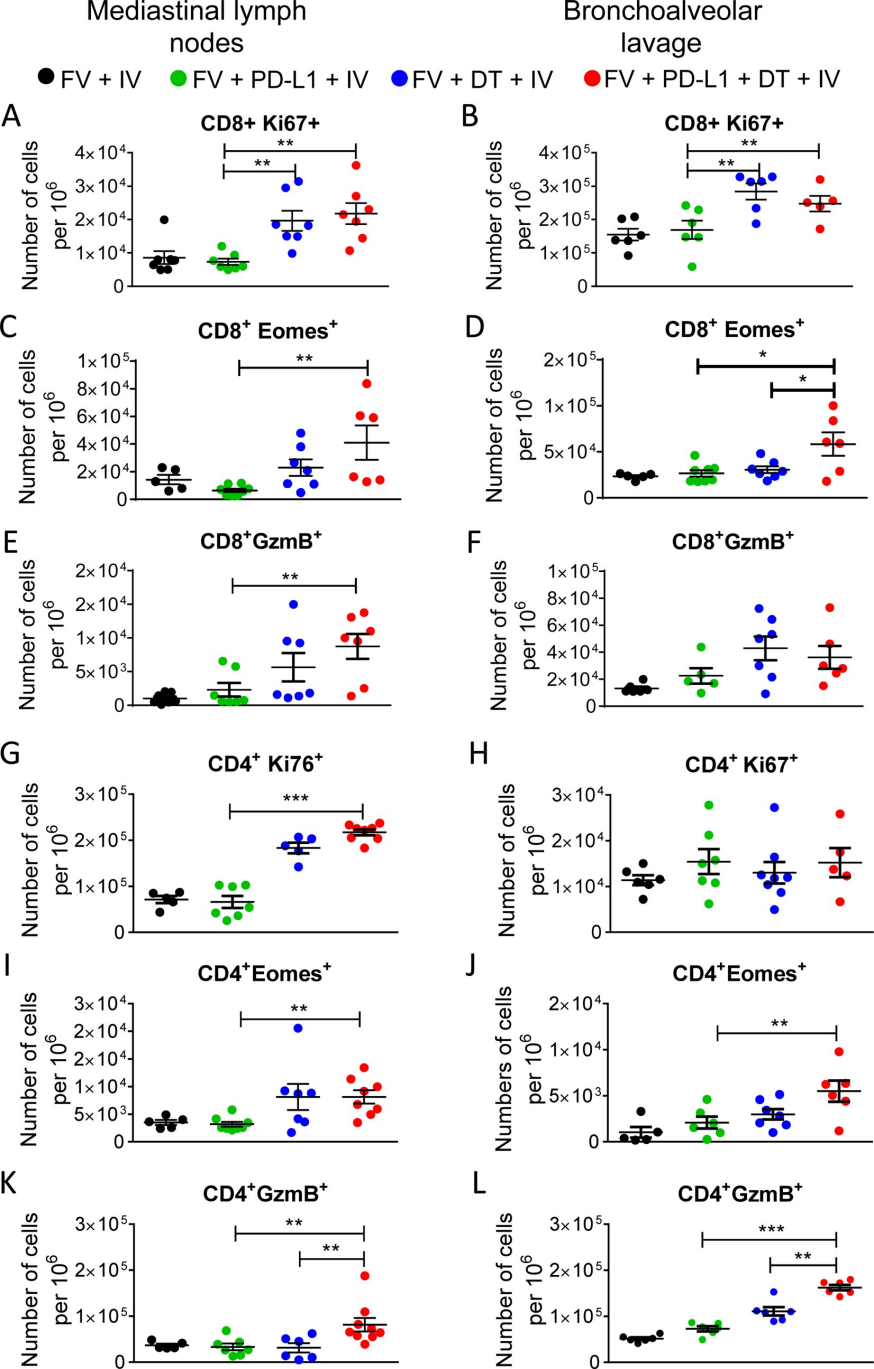
Characterization of CD8+ T cells and CD4+ T cells isolated from mediastinal lymph nodes and from bronchoalveolar lavage. Mice chronic infected with FV were treated with DT (DEREG mice) and/or blocking antibodies against PD-L1. One day after the start of treatment mice were infected with IV and, 12 days after IV infection, were killed and mediastinal LN and BALF were analyzed by flow cytometry. Frequencies of CD8+ T cells positive for Ki67+ in MLN (**A**) and in BALF (**B**), numbers of CD8+ T cells positive for Eomes in MLN (**C**) and in BALF (**D**), and numbers of CD8+ T cells producing GzmB in MLN (**E**) and in BALF (**F**). Frequencies of CD4+Foxp3- T cells positive for Ki67 in MLN (**G**) and in BALF (**H**), numbers of CD4+Foxp3- T cells positive for Eomes in MLN (**I**) and in BALF (**J**), and numbers of CD4+Foxp3- T cells producing GzmB in MLN (**K**) and in BALF (**L**). Each dot represents an individual mouse. Data were pooled from 2 to 4 independent experiments with similar results. Statistically significant differences are indicated by asterisks (* < 0.05; ** < 0.005; *** < 0.0005; one-way ANOVA with a Tukey post-test).

To further define the role of T cells in the observed immunopathology of the lung after IV infection, we investigated the phenotype and functional properties of CD8+ and CD4+ T cells isolated from mediastinal lymph nodes (mLN) or from BALF. Both CD4+ as well as CD8+ T cells strongly proliferated (expressed Ki67) in mice that were IV-infected and received Treg depleting DT or combination therapy (Fig 6A, 6B, 6G and 6H). This was mainly found in mLN but also for cells from BALF of some mice. In addition, we also stained T cells for the surrogate markers of cytotoxicity GzmB and Eomes in mLN and BALF. The numbers of T lymphocytes expressing markers of cytotoxicity were highest in Treg depleted IV-infected mice as well as in animals that received combination therapy (Fig 6C–6L). Overall, the highest numbers of T cells expressing GzmB or EOMES were found in mice treated with the combination therapy, which was in many cases significantly higher than the numbers in mice receiving either treatment alone. Thus, the lung pathology in mice was associated with the expansion of CD4+ and CD8+ T cells with cytotoxic potential in mLN and in lung tissue.

**Fig 7:**
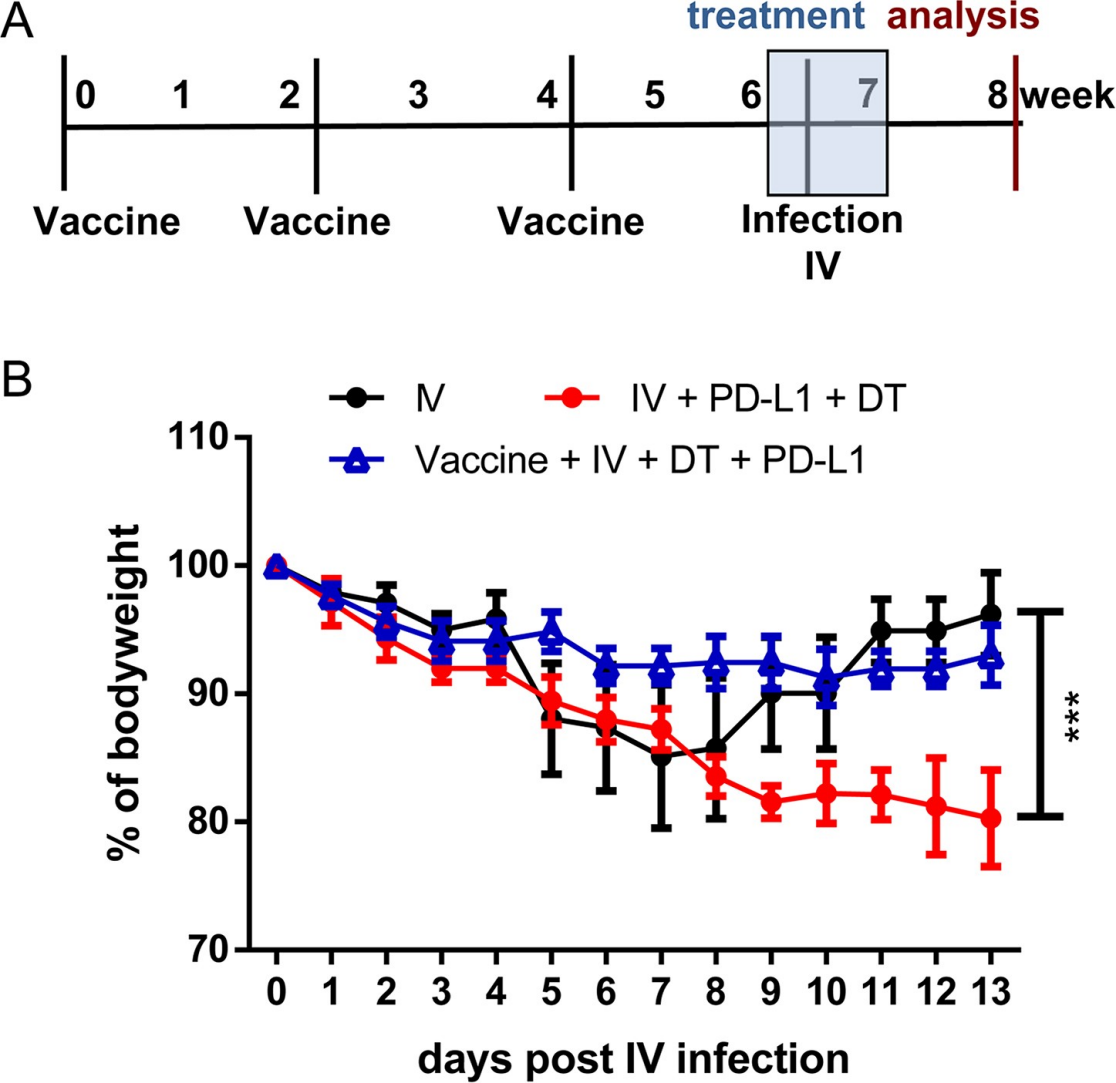
Vaccination prevents the development of immune-mediated pathology. DEREG mice were immunized with an inactivated influenza virus and treated with DT and anti-PD-L1 antibody (**A**). Weight reduction of mice infected with IV (black line), IV-infected and treated (blue line), and vaccinated, IV-challenged and treated (red line) (**B**). Data were pooled from 2 independent experiments with similar results. Statistically significant differences are indicated by asterisks (*** < 0.0005; non-parametric Mann-Whitney test).

The current findings demonstrate that the infection of mice with a low dose of IV resulted in severe immunopathology of the lung under immune checkpoint blocking therapy. Vaccination against IV prevents severe symptoms after influenza infection in humans. In order to test whether a vaccination would also prevent the development of immune-mediated IV-induced pathology after combination therapy in mice, the following experiment was proposed. Naïve mice were vaccinated three times with inactivated IV every two weeks (Fig 7A**)**. Two weeks after the last immunization combination treatment was started. On the following day mice were infected with IV and their weight was followed to monitor the development of pathology (Fig 7B). These experiments were performed without previous FV infection in both the vaccinated as well as the non-vaccinated control group. IV-infected mice without treatment showed transient weight loss between day four and day ten after infection, but fully recovered. Loss of weight in the group of mice with combination therapy also started on day four and progressed until the animals had to be euthanized on day 13. In contrast, vaccinated mice did not show any significant reduction of body weight under IV infection and therapy. Thus, pre-therapy vaccination against IV may be an option to reduce the risk of virus-induced immunopathology during immune checkpoint therapy.

### Cytotoxic CD4+ and CD8+ T cells in the peripheral blood of melanoma patients developing intestinal autoimmune pathologies under immune checkpoint combination therapy

**Fig 8:**
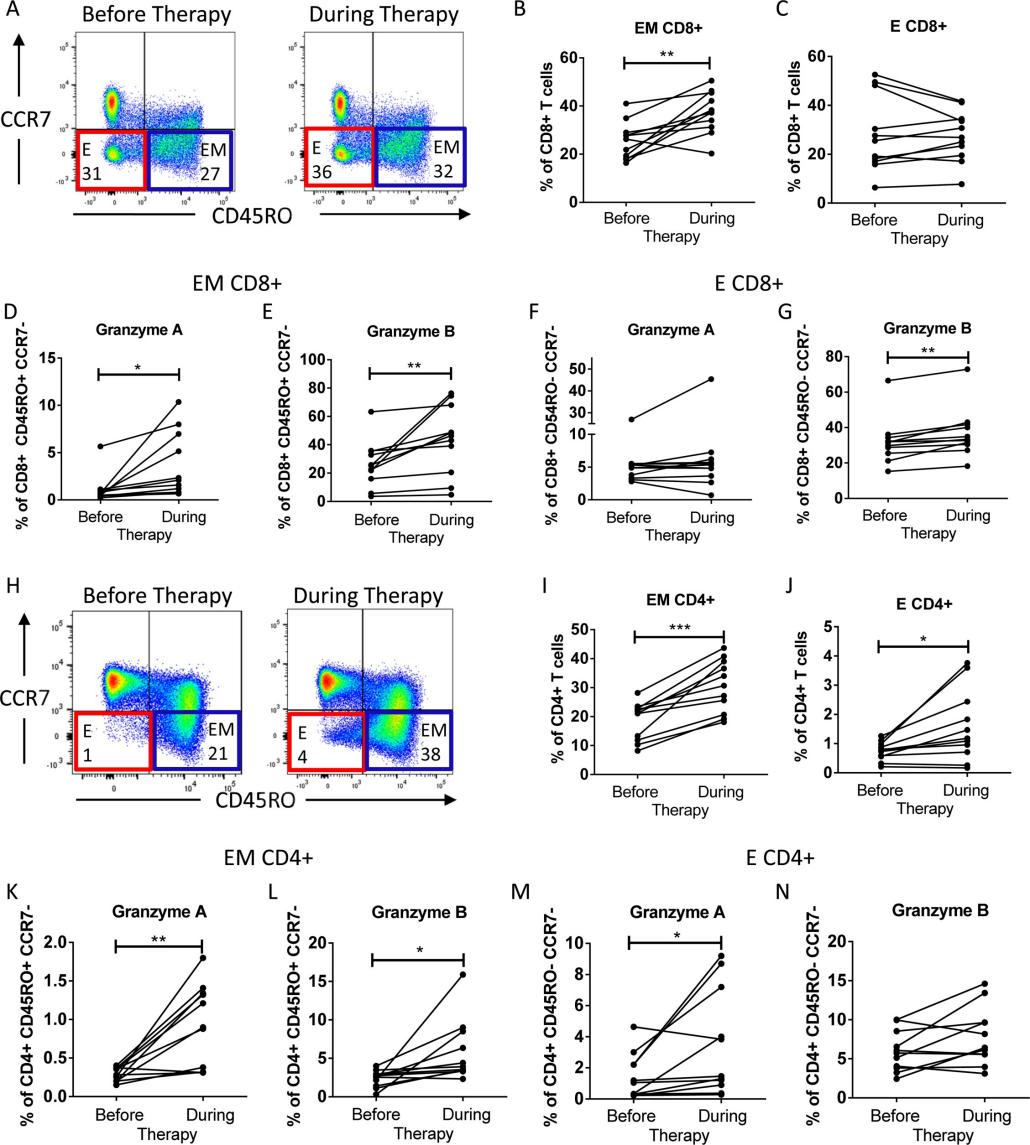
Characterization of CD8+ T cells and CD4+ T cells from PBMCs of melanoma patients with symptoms of AiRE after checkpoint blocking therapy. Multiparameter flow cytometry was used for the analysis of CD8+ and CD4+ T cell differentiation and production of GzmA and GzmB. Representative dot plots of CD8+ T cells of a melanoma patient before combination therapy and during the therapy (**A**). Percentage of CD8+ T cells with the phenotype of effector-memory (EM) (CD45RO+ CCR7-) (**B**) or effector cells (E) (CD45RO- CCR7-) (**C**). Percentage of EM CD8+ produced GzmA (**D**) or GzmB (**E**); percentages of E CD8+ producing GzmA (**F**) or GzmB (**G**). Representative dot plots of CD4+ T cells of a melanoma patient before combination therapy and during the therapy (H). Percentage of CD4+ T cells with the phenotype of EM (CD45RO+ CCR7-) (I) or E (CD45RO- CCR7-) (J). Percentage of EM CD4+ T cells producing GzmA (**K**) or GzmB (**L**); percentages of E CD4+ T cells producing GzmA (**M**) or GzmB (**N**). Each dot represents data from an individual patient. Data from the same patient are connected. Statistically significant differences are indicated by asterisks (* < 0.05; paired nonparametric t-test).

Our results from the combination therapy in acutely infected animals provide evidence that immunopathology was caused by expanded CD4+ and CD8+ T cells that produced granzymes. Immune-related adverse events (IrAE) are the main complication during the treatment of different malignancies with checkpoint blockers. Interestingly, treatment with anti-CTLA-4 antibodies was reported to deplete Tregs, which express high levels of CTLA-4 [43,44]. Thus, the treatment of melanoma patients with a combination of nivolumab (anti-PD-1 antibody) and ipilimumab (anti-CTLA-4 antibody) seems to have similar immunological effects as our combination therapy described above. Moreover, about 95.5% of patients receiving combination treatment develop IrAE symptoms of different grades [45]. To study this in more detail, PBMC samples of eleven melanoma patients were obtained before combination therapy with nivolumab and ipilimumab and after the manifestation of gastrointestinal IrAE symptoms (five ± two week after therapy start). The clinical data of the analyzed patients are summarised in S3 Table. Most patients (eight out of eleven) had disease control with complete, partial, stable or mixed responses to therapy. However, the development of severe IrAE symptoms led to premature termination of the combination therapy with ipilimumab and nivolumab in eight patients. Thereafter, patients were treated with corticosteroids alone or in combination with additional immunosuppressive drugs. No significant changes in the frequencies of lymphocytes or myeloid cells were associated with the development of IrAE during combination therapy (S3 Table). The phenotype of CD4+ and CD8+ T cells and the intracellular expression of GzmA and GzmB in these cells were analyzed before therapy and at the manifestation of therapy-induced symptoms (IrAE). The frequency of CD8+ T cells with an effector memory (CD45RO+ CCD7-) phenotype was significantly enhanced after treatment (Fig 8A and 8B). The frequency of effector CD8+ T cells (CD45RO- CCR7-) was also enhanced after treatment in seven out of eleven investigated patients (Fig 8A and 8C). In addition, the percentage of effector memory CD8+ T cells expressing GzmA or GzmB was significantly increased (Fig 8D and 8E). In the population of effector CD8+ T cells only the production of GzmB was enhanced (Fig 8F and 8G). The populations of circulating CD4+ T cells with effector memory or effector phenotype were significantly expanded in all investigated patients under treatment (Fig 8H–8J). The frequencies of CD4+ T cells producing GzmA and GzmB in both effector T cell subpopulations were enhanced (Fig 8K–8N), but in the population of effector CD4+ T cells differences for GzmB were not significant. Thus, similar to our animal experiments, a combination therapy in melanoma patients led to the expansion of effector CD4+ and CD8+ T cells showing enhanced production of granzymes.

## Discussion

An acute virus infection strongly activates the immune system and multiple inhibitory mechanisms are necessary to counterbalance this response in infected organs or even systemically. Tregs and inhibitory receptors are the two most powerful controllers of the activation and function of T lymphocytes in infectious diseases [8,46]. Both of these inhibitory mechanisms work independently of each other and suppress the differentiation and expansion of cytotoxic CD8+ T cells during chronic infection. Thus, the combination of depletion/inhibition of Tregs together with the blockage of inhibitory receptors results in a strong enhancement of antiviral T cell responses and was proposed as a treatment of chronic infections or cancers [28]. However, if this treatment was performed during acute viral infections it resulted in severe immunopathology and death of treated animals. The lethality was associated with a systemic activation and expansion of cytotoxic CD8+ and CD4+ T cells. Immune responses against pathogens are regulated by different stimulatory and inhibitory pathways to reach a balance between the elimination of virus-infected cells and the prevention of immunopathology, which may result in the failure of infected organs. The recognition of molecular viral patterns by infected and innate immune cells induces several mechanisms mobilizing the immune system [47] including the secretion of proinflammatory cytokines and type I IFNs. The expansion, functional differentiation and apoptosis of stimulated T cells during an acute infection are strictly controlled by different immunomodulatory mechanisms. Tregs and inhibitory receptors are important players of this immune regulation [48–50]. This regulation is also involved in the selection of T cells with high antiviral specificity. In our current experiments, numbers of virus-specific CD4+ and CD8+ T cells only moderately increased after combination therapy during acute FV infection, at least those for the two immunodominant T cell epitopes we analyzed. Thus, improved functional properties of T cells due to immune checkpoint blockers seem to be more important for the observed development of immunopathology in mice. However, an acute virus infection was required to tip the balance towards immunopathology during an ongoing combination immune checkpoint blocking therapy. Is this a problem that cancer patients undergoing combination therapy face? Up to now there is no published data on frequencies and consequences of acute infections in such patients. However, the most successful combination checkpoint blocking therapy in cancer patients is very similar to what we did in our mouse studies. Combination treatment with antibodies against CTLA-4 and PD-1/PD-L1 was very potent in inducing tumor regression [51]. It was shown that the anti-CTLA-4 antibodies, currently used for the treatment of melanoma and lung cancer patients, significantly reduce the numbers of Tregs [52,53], and it is discussed if this is the mechanism how they actually activate the anti-tumor immune response. Interestingly, the main complications during the treatment of patients with PD-1/PDL-1 blocking antibodies and with Treg-reducing CTLA-4 antibodies [28,29] are autoimmune disorders that are frequently manifested in the gut [51]. These disorders, also referred to as immune-related adverse events (irAE), are the main indication for termination of treatment with immune checkpoint blockers. In some cases, combination therapy led to irAE with fatal outcome [54]. It is possible, but not so far not documented, that seasonal acute viral infections may have contributed to severe irAE pathology. Our mouse data suggest that prevention or rapid control of acute infections might be important in cancer patients undergoing combination therapy with several immune checkpoint inhibitors. One obvious difference between our mouse data and the situation in human patients is that the anti-CTLA-4 antibody does only result in a partial depletion of Tregs in humans [29], whereas DT injection into DEREG mice results in a complete Treg depletion that lasts at least for several days. This might induce more severe (lethal) pathology in the mouse model after an acute infection during combination therapy compared to human patients. In the future, the population of Tregs may be also be affected by other immunotherapies that are currently tested in clinical trials or preclinical experiments. Antibodies specific for CCR4 (C-C chemokine receptor type 4), GITR (glucocorticoid-induced TNF-receptor family related protein), or OX40 (Tumor necrosis factor receptor superfamily, member 4, CD134) [55] all reduce Treg responses. Moreover, some conventional chemotherapeutics for the treatment of different types of cancers (summarized in review [56]) also reduce the numbers of Tregs and/or the functionality of these cells. The combination of such drugs with immune checkpoint blockers may therefore also induce the risk of severe pathology during an acute infection.

In the main part of this study we used a murine virus to expore immunopathology during immune checkpoint therapy. In order to investigate whether a similar pathology develops under combination therapy after infection with a human virus we inoculated mice with low doses of IV. These animals were chronically infected with FV to characterize how a successful therapy against a chronic infection tips towards immunopathology under the condition of an acute virus infection. In these experiments, animals that were depleted for Tregs as well as those receiving combination therapy developed severe pneumonia even though IV replication was efficiently controlled. In the case of IV, the pathology was mainly observed in the lung, the main target organ of viral replication and subsequent antiviral immune responses. Again, the complete depletion of Tregs in the DEREG model might have contributed to the severe pathology after single immunotherapy. However, also in this model maximum immune activation was found in mice receiving combination therapy. It was recently shown that Tregs in the lung of IV infected mice did not only have a suppressive activity on effector T cells [57] but were also necessary for the repair of lung tissue damaged by the virus. The elimination of Tregs potentiated the damage of the lung and the severity of lung pathology after IV [57] infection, but also during Respiratory Syncytial Virus (RSV) infection [58,59]. This suggests that Tregs might be especially important in protecting lung tissue during an acute infection and that immunotherapies targeting Tregs might pose a special risk for severe lung infections. Interestingly, the IV vaccination of mice prior to immunotherapy and IV challenge prevented the development of lethal immunopathology (Fig 7B). Thus, vaccination may be an important prophylactic option to prevent virus-mediated T cell activation and the development of irAE complications during immunotherapy. Contradictory results from two recent studies which analyzed the effects of IV vaccination during anti-PD-1 therapy of patients with lung cancer were published [60, 61]. In one study an enhanced frequency of irAE after IV vaccination was observed [60], while another study reported no differences between vaccinated and non-vaccinated patients [61]. These studies only investigated the direct effects of vaccination but not the possible protection from infection. However, our data suggest that vaccination prior to immunotherapy might be more appropriate to prevent pathologies than during therapy.

Another approach might be the prophilactic neutralization of TNF-α to reduce the symptoms of irAE during checkpoint blockade [62]. The enhanced production of different cytokines including TNF-α was also observed in our study (Fig 4E) which suggests that anti-TNF-α treatment might prevent the development of pathology. Type I Interferon responses are also often associated with immunopathology[63], but so far no data exists to answer the question if they contribute to irAE during checkpoint therapy.

The immunopathology seen in our mouse experiments was likely associated with the expansion of cytotoxic T cells. Previously it was shown that expanded LCMV-induced CD8+ T cells were responsible for the development of immunopathology in mice [64]. The exhaustion of such cells is a possible mechanism to reduce pathology in infected organs, as they can be very harmful for many host cells and have to be tightly controlled during an ongoing immune response. In particular, the massive expansion of cytotoxic CD4+ T cells was frequently reported to be associated with immunopathology. An accumulation of cytotoxic CD4+ T cells was observed in patients with multiple sclerosis (MS) [65,66], inflammatory bowel disease (IBD) [67], rheumatoid arthritis (RA) [68], and ankylosing spondylitis [69]. Interestingly, Human Parvovirus B19 specific GzmB producing CD4+ T cells may contribute to RA and systemic lupus erythematosus (SLE) associated with chronic B19 infection [70]. Thus, CD4+ T cell cytotoxicity seems to be a hallmark of immunopathology. During acute FV infection, the CD4+ T cell cytotoxicity is controlled/suppressed by Tregs [34]. Also immune checkpoint receptors have been shown to regulate the differentiation of cytotoxic CD4+ T cells [71]. This implies that CD4+ cytotoxicity is be tightly controlled because these cells have the potential to induce severe damage in infected or even healthy tissue. Our current data indicate that several different immune checkpoints contribute to this control.

Taken together, seasonal viral infections circulating in the human population are often asymptomatic or induce minimal symptoms. However, an acute infection of cancer patients undergoing combination therapy may provoke a systemic activation of the immune system with progression to immunopathology. Another unanswered question is whether latent human viruses, like CMV, EBV, HSV may contribute to the development of immunopathology during the treatment with checkpoint blockers. One study actually reported a reactivation of latent CMV during checkpoint blocking therapy [72]. The observations described in the current study provide a warning for current and future treatments targeting regulatory immune mechanisms, especially those affecting Tregs. Enhanced numbers of GzmB producing CD4+ and CD8+ T cells in tumor patients after combination treatment with antibodies against PD-1 and CTLA-4 have previously been described [73], but this study did not perform a detailed characterization of the GzmB producing cells. Our data indicate that T cells producing granzymes during combination therapy express an effector or effector-memory phenotype. The expansion of these cells was timely associated with the appearance of irAE symptoms. Although our human study does not provide a causative link between granzyme-producing CD4+ or CD8+ T cells and irAE, we can speculate that these CTLs might be potentially harmful and their responses may be accelerated during virus infections. This hypothesis was recently supported by a case report of fatal encephalitis that developed after anti-PD-1 therapy in a melanoma patient [74]. The injured regions of the patient’s brain showed a massive expansion of EBV-specific CD4+ T cells producing GzmB. Based on these data, it can be important to monitor the numbers of effector and effector-memory CD4+ and CD8+ T cells and their production of GzmB during the treatment with immune checkpoint blockers. Another practical conclusion from the current data is the necessity to protect patients from acute viral infections by vaccination before treatment is started.

## Methods

### Ethics statement

Animal experiments were performed in strict accordance with the German regulations of the Society for Laboratory Animal Science (GV-SOLAS) and the European Health Law of the Federation of Laboratory Animal Science Associations (FELASA). The protocol was approved by the North Rhine-Westphalia State Agency for Nature, Environment and Consumer Protection (LANUV). All efforts were made to minimize suffering. Samples from adult patients were collected after written informed consent. Studies on human material were approved by the institutional review board of the University of Duisburg-Essen.

### Mice

Inbred C57BL/6 (B6) and DEREG [30] mice were maintained under pathogen free conditions. Experiments were done using mice (H-2^b/b^, Fv1^b/b^, Fv2^r/r^) on C57BL/6 background that are resistant to FV-induced leukemia. All mice were females of 8–12 weeks of age at the beginning of the experiments.

### Virus and viral infection

The FV stock used in these experiments was FV complex containing B-tropic Friend murine leukemia helper virus (F-MuLV) and polycythemia-inducing spleen focus-forming virus [75]. The stock was prepared as a 10% spleen cell homogenate from BALB/c mice infected 14 days previously with 3,000 spleen focus-forming units of non-cloned virus stock. Experimental mice were injected intravenously with 20,000 spleen focus-forming units of FV complex.

Mice, which were at least 30 days infected with 20,000 spleen focus-forming units of FV complex, were anesthetized by ketamine/xylazin (25μl) and intranasally infected with 15 PFUs of influenza virus A/PR/8/34.

### Inactivation of influenza virus

A high-titered (10^9 pfu/ml), chicken egg-grown stock of PR/8/34 was treated for 24 h with 0.1% β-propiolactone (Acros Organics) at room temperature, followed by dialysis for 24 h against HNE buffer (5 mM HEPES, 150 mM NaCl, 0.1 mM EDTA, pH 7.4) at 4° C.

### Immunization of mice and IV challenge

Mice were immunized i.p. with 1μg of inactivated IV diluted in 300 ml PBS on days 0, 14, and 28. On day 42 after initial immunization, mice were anesthetized by ketamine/ xylazine and challenged intranasally with 15 PFUs influenza virus A/PR/8/34 (50 ml).

### In vivo blockade

For blockade of the PD-1 pathway in acute FV-infected mice, 200μg rat anti-mouse PD-L1 Ab (10F.9G2; BioXCell) was administered intraperitoneally every third day for 3 times. To block the Tim-3 pathway, 100μg rat anti-mouse Tim-3 Ab (RMT3-23; BioXCell) was administered intraperitoneally every other day for 4 times (Fig 1A). The T cell responses and viral loads were analyzed one day post treatments or 4 days post treatments (Fig 1A).

During chronic FV infection mice were treated with 200μg rat anti-mouse PD-L1 Ab (10F.9G2; BioXCell) intraperitoneally every third day for 3 times.

### Re-directed killing assay

Measurement of the cytotoxic activity of CD4+ and CD8+ T cells was performed by using a previously described re-directed killing assay [39]. CD4+ and CD8+ T cells isolated with magnetic beats were co-incubated for 5 hours with anti-mouse CD3 antibody-loaded P815 target cells. After incubation the activity of LDH in the supernatant was measured using Pierce LDH Cytotoxicity Assay Kit (Thermo Sciantific). The percentage of target cell elimination was calculated as % cell lysis = (experimental–effector spontaneous–low control) x 100/ (high control–low control). Cytotoxicity assays were performed with varying effector cell (E) to target cell (T) ratios, and percentages of lysis are reported as the mean ± SD of triplicate samples.

### Infectious center assays

Infectious center assays were performed as described previously [76].

### Determination of lung influenza virus copy number

Lungs were collected on day 12 after IV challenge; they were homogenized in PBS containing 0.3% BSA and centrifuged for 8 min. Viral RNA was isolated from 140 μl lung homogenates using the QIAamp Viral RNA Mini Kit (Qiagen) according to the manufacturer’s instructions. The quantity of viral RNA was determined by real-time RT-PCR (RT-quantitative PCR) using the QuantiTect Probe RT-PCR Kit (Qiagen) with SYBR-Green. A conserved sequence in the matrix gene of influenza A virus was detected using the specific primers described elsewhere (5′-CTTCTAACCGAGGTCGAAACG-3′ + 5′-AGGGCATTTTGGACAAAG/TCGTCTA-3′) (19).

### Isolation of lamina propria lymphocytes from the small intestine

Lamina propria lymphocytes were isolated from small intestines as described previously [77]. In brief, small intestines were rinsed extensively with PBS, and residual mesenteric fat tissue and Peyer`s patches were resected. Intestines were cut open longitudinally and sliced into small pieces. Under constant stirring at 37°C, the intestines were incubated twice in PBS containing 3 mmol/L EDTA. EDTA was removed by incubating the intestines twice in RPMI-1640 medium containing 1% FCS, 1 mmol/L EGTA, and 1.5 mmol/L MgCl2. Small intestines were digested in RPMI-1640 containing 20% FCS and 100 U/mL collagenase IV (Sigma-Aldrich) at 37°C for 30 minutes. To obtain single cells, the remaining suspensions were passed through 30-μm cell strainer and washed with RPMI medium.

### Immunohistochemistry of intestine

Immunofluorescence analyses were performed on snap-frozen tissues. Intestine sections were stained for CD8 T cells (anti-CD8; company, clone 53–6.7, eBiosciences), CD4 T cells (anti-CD4; clone RM4-5, eBiosciences)). Scale bar indicates 100μm, as indicated in the figure legend.

### Cell surface and intracellular staining by flow cytometry

Surface and intracellular staining were performed as described previously [20]. For surface staining were used antibodies specific against mice CD3 (17A2), CD4 (RM4-5), CD8 (53–6.7), CD11a (M17/4) CD43 (1B11), CD44 (IM7), CD62L (MEL-14), CD69 (H1.2F3), CD127 (AFR134), KLRG1 (2F1), and for intracellular staining were used antibody specific against mice EOMES (Dan11mag), Foxp3 (FJK-16S), GzmB (GB11), Ki67 (B56), and T-bet (4B10) produced by eBiosciences or BioLegend. For surface staining of human cells were used antibodies specific against human CD3 (SK7), CD4 (OKT4), CD8 (HIT8a), CD45RO (UCHL1), CCR7 (G043H7), and for intracellular staining were used antibody specific against human GzmA (CB8) and GzmB (GB11) produced by BioLegend.

Data were acquired on a LSR II flow cytometer (Becton Dickinson) from 350,000–500,000 lymphocyte-gated events per sample. Analyses were done using FACSDiva software (Becton Dickinson) and FlowJo software (Treestar).

### Cytokine staining

Cytokine levels were estimated using the LegendPlex (BioLegend) and ProcartaPlex Mouse Th1Th2 essential 6 plex (EPX060-20831-S1, Affymetrix), following manufacturers instructions. The ProcartaPlex assay was measured by Luminex, using Luminex IS software.

### Lymphocyte depletion

To deplete Tregs, FV-infected DEREG mice were injected intraperitoneally with diphtheria toxin (Merck, Darmstadt, Germany), diluted in endotoxin-free PBS. 0.5 μg DT was inoculated every third day for 3 times. CD4+ and CD8+ T cell depletions were performed essentially as described previously [50]. Mice were inoculated three times every other day, starting on day 12 after FV infection. The treatment depleted 92% of CD4+ T cells and 87% of CD8+ T cells.

### Tetramers and tetramer staining

For detection of D^b^-GagL-specific CD8^+^ T cells, nucleated spleen cells were stained with PE labeled MHC class I H2-D^b^ tetramers specific for FV GagL peptide [33,78] (Beckman Coulter, Marseille, France).

MHC class-II tetramer (Tet II) A^b^-restricted F-MuLV-envelope epitope (H19-Env) (EPLTSLTPRCNTAWNRLUL) staining of FV-specific CD4+ T cells was performed as previously described [35].

For detection of CD8^+^ T cells specific to the nucleoprotein (NP_366-374_) of IV the staining with PE labeled H2Db NP_366-374_ tetramers was performed [79,80].

### Label-free quantitative mass spectrometry

Cells were lysed by sonication (6 10-s pulses on ice) in sample buffer (50 mM NH4HCO3; 0.1% Rapigest). Protein isolation, digestion, amino acid analysis, and liquid chromatography-tandem mass spectrometry (LC-MS/MS) analysis were performed by following previously described procedures [81]. Progenesis QI for proteomics (v. 2.0.5387.52102; Non-linear Dynamics, Newcastle upon Tyne, UK) and Proteome Discoverer (v. 1.4; Thermo Scientific, Bremen, Germany) software packages were used for protein quantification and identification, respectively. For protein identification, LC-MS/MS runs were searched against the UniProt-SwissProt database (Release 2014_10; v. 2.5; 546,790 sequences) with taxonomy restriction to *mus musculus*. Further search parameters matched previously reported ones [81]. Non-unique peptides associated to more than one protein accession were not used for quantification. Differentially expressed proteins (p < 0.05) were functionally annotated using the Database for Annotation, Visualization, and Integrated Discovery (DAVID, ver. 6.8) [82,83].

### Statistical analysis

Statistics comparing the two groups were done using the unpaired non-parametric t-test or Mann-Whitney t-test. When more than two groups were compared, a one-way ANOVA was used with a Tukey post-test. (GraphPad Prism software; GraphPad Software Inc., San Diego, USA). For statistical analysis of proteomics data, normalized protein abundances were exported from Progenesis QI software and transformed using arcsinh-function. Using an in-house written R script (R Foundation for Statistical Computing, Vienna, Austria), Benjamini–Hochberg corrected one-way ANOVA was used for the calculation of the FDR-corrected p-values as described earlier [81].

## Supporting information

S1 Fig Representative dot plots of CD8+ and CD4+ T cells after combination therapy. DEREG mice were infected with FV and treated with DT and/or blocking antibodies against PD-L1 and TIM-3. Flow cytometry was used for the determination of spleen CD8+ T cells which are expressing CD43 (A), positive for MHC class I H2-Db tetramers specific for FV GagL peptide (Tetr+) (B), and producing the GzmB (C), and the percentages of CD4+ T cells producing the GzmB (D). Representative dot plots of one mice per every group are presented.
(TIF)

S2 Fig Inflammation in liver, pancreas, and stomach. Hematoxylin and eosin staining of paraffin sections of liver, pancreas, and stomach of mice with combined DT and PD-L1/Tim3 treatment and control mice. The images were captured at 40x magnification (liver and pancreas) and 20x magnification (stomach).
(TIF)

S3 Fig Immunofluorescence analysis of intestine sections. C57BL6 (groups of FV infected mice without treatment and mice with PD-L1/Tim3 treatment) and DEREG (groups with DT treatment and group of mice with combined DT and PD-L1/Tim3 treatment were infected with FV and were treated with DT and/or blocking antibodies against PD-L1 and TIM-3 as indicated during the second week of infection. The intestine sections were stained for DAPI (blue), CD4+ T cells (red), and CD8+ T cells (green). Fluorescent images were captured at 20x magnification using KeyenceBZ-9000E microscope.
(TIF)

S4 Fig Characterization of CD8+ T cells and CD4+ T cells isolated from inguinal lymph nodes. Mice were infected with FV and were treated with DT and/or blocking antibodies against PD-L1 and TIM-3 as indicated (Fig 1A). 18 days after infection mesenteric lymph nodes were isolated and the flow cytometry analysis of CD8+ and CD4+ T cells was performed. Mean percentages of CD8+ T cells (**A**) and CD4+ T cells (**B**) expressing T-bet, CD43, CD44, CD11a, KLRG1, Ki67, CD69, or negative for CD62L and for CD127 from 5–8 mice are presented. Data were pooled from 2 or 3 independent experiments with similar results.
(TIF)

S1 Table Global proteome analysis of expanded CD4+ and CD8+ T cells. Mice were infected with FV and were treated with DT and blocking antibodies against PD-L1 and TIM-3 as indicated (Fig 1A). At 18 days post infection CD3+CD8+CD43+ T cells and CD3+CD4+CD43+CD62L- T cells were sorted from the spleens of FV-infected DEREG mice and from infected DEREG mice treated with DT plus anti-PD-L1/Tim-3 antibodies. Cells were lysed and subjected to proteome analysis performed by label-free quantification using liquid chromatography and tandem mass spectrometry (LC-MS/MS).
(XLSX)

S2 Table Clustering analysis of differently expressed proteins. Mice were infected with FV and were treated with DT and blocking antibodies against PD-L1 and TIM-3 as indicated (Fig 1A). At 18 days post infection CD3+CD8+CD43+ T cells and CD3+CD4+CD43+CD62L- T cells were sorted from the spleens of FV-infected DEREG mice and from infected DEREG mice treated with DT plus anti-PD-L1/Tim-3 antibodies. Cells were lysed and subjected to proteome analysis performed by label-free quantification using liquid chromatography and tandem mass spectrometry (LC-MS/MS). Differently expressed proteins were analyzed with the Gene Ontology enrichment tool (GO analysis).
(XLSX)

S3 Table Clinical data of patients. Clinical data of a melanoma patients treated with a combination of nivolumab (anti-PD-1 antibody) and ipilimumab (anti-CTLA-4 antibody).
(XLSX)
