# Supplementary figures and images for: Combination immunotherapy with anti-PD-L1 antibody and depletion of regulatory T cells during acute viral infections results in improved virus control but lethal immunopathology
Source: PLoS Pathog. 2020 Mar 30;16(3):e1008340. doi: 10.1371/journal.ppat.1008340 (PMC7105110; doi:10.1371/journal.ppat.1008340)

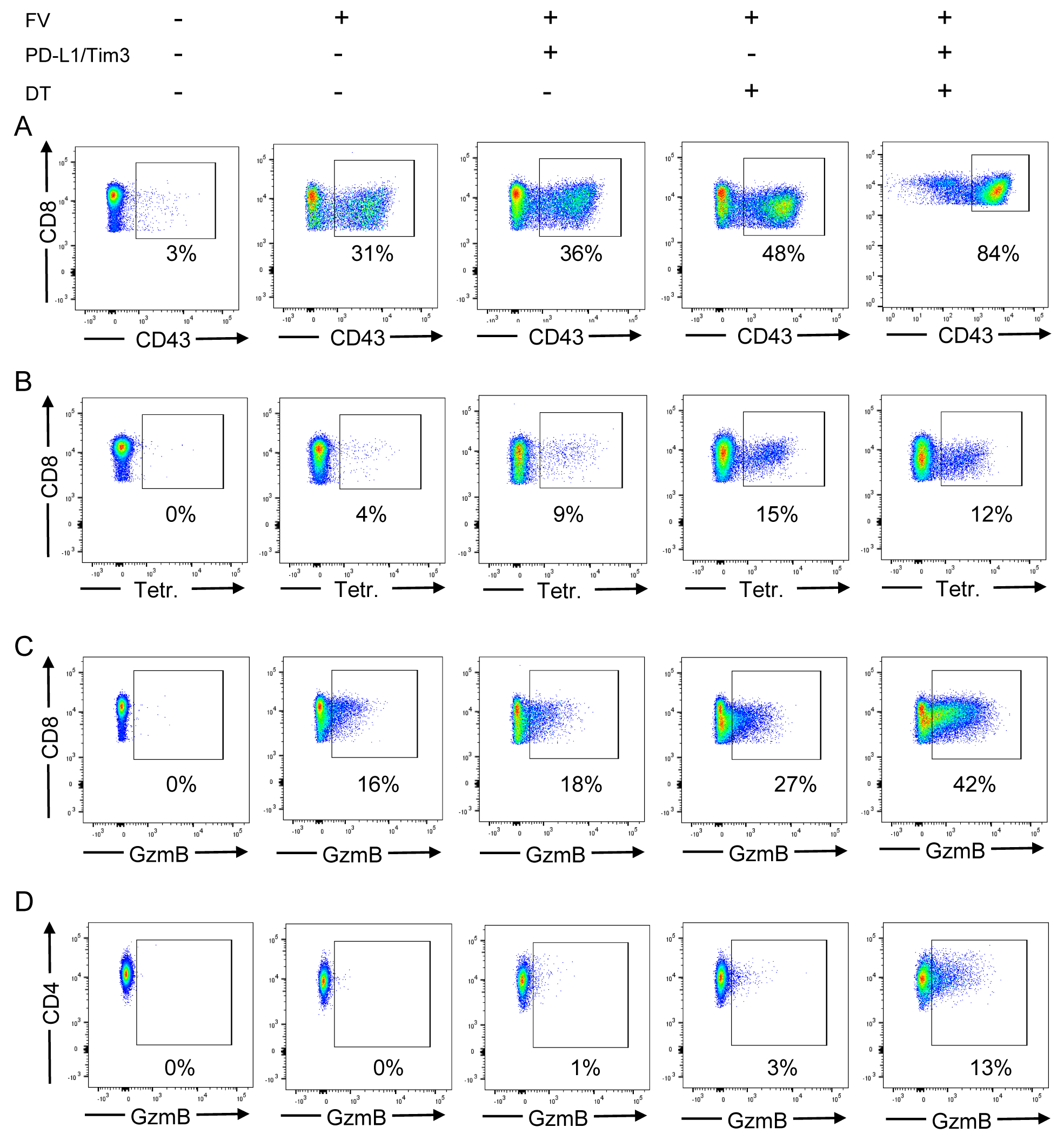

Supplement: S1 Fig — DEREG mice were infected with FV and treated with DT and/or blocking antibodies against PD-L1 and TIM-3. Flow cytometry was used for the determination of spleen CD8+ T cells which are expressing CD43 (A), positive for MHC class I H2-Db tetramers specific for FV GagL peptide (Tetr+) (B), and producing the GzmB (C), and the percentages of CD4+ T cells producing the GzmB (D). Representative dot plots of one mice per every group are presented. (TIF) [file ppat.1008340.s001.tif]

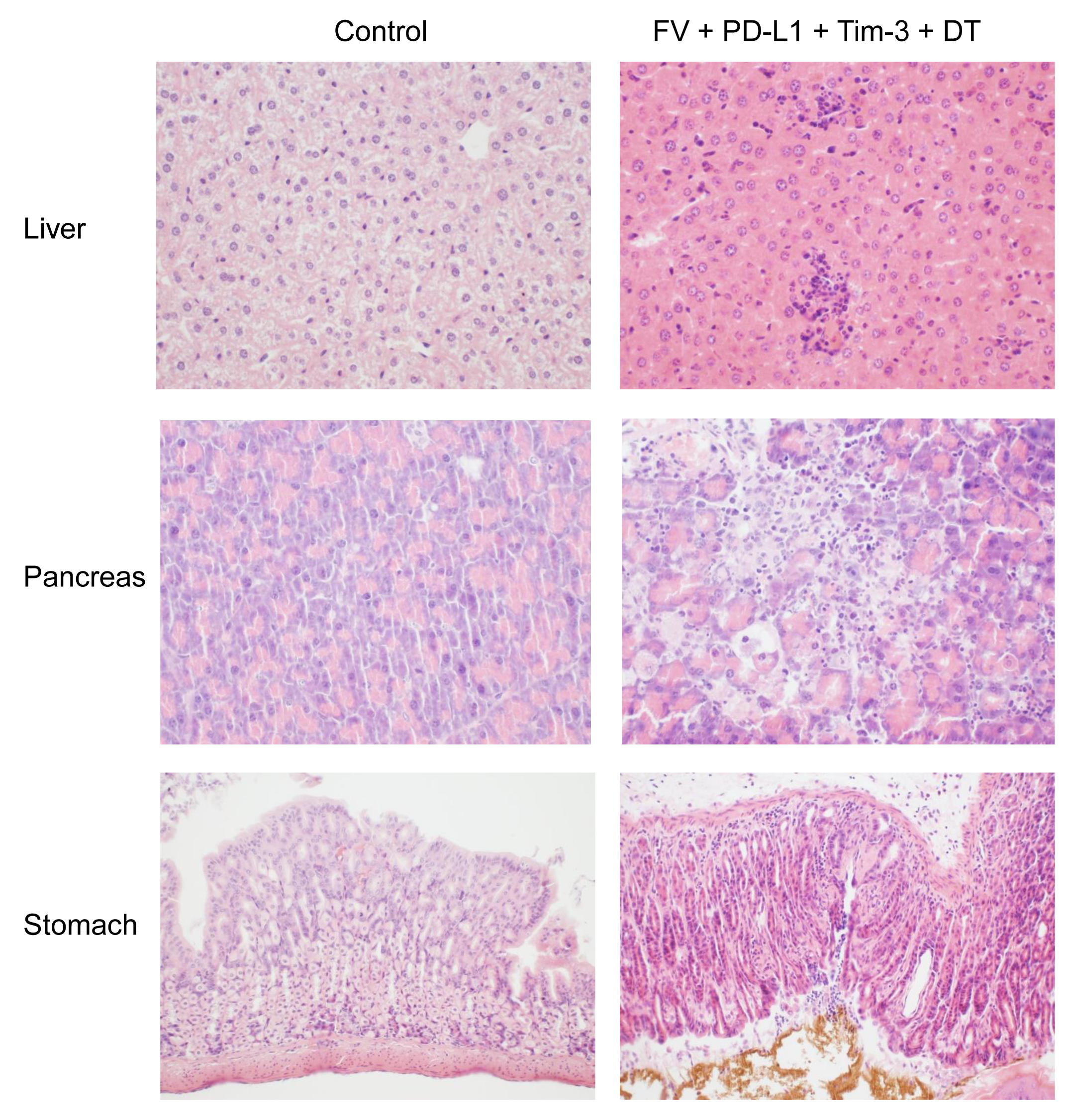

Supplement: S2 Fig — Hematoxylin and eosin staining of paraffin sections of liver, pancreas, and stomach of mice with combined DT and PD-L1/Tim3 treatment and control mice. The images were captured at 40x magnification (liver and pancreas) and 20x magnification (stomach). (TIF) [file ppat.1008340.s002.tif]

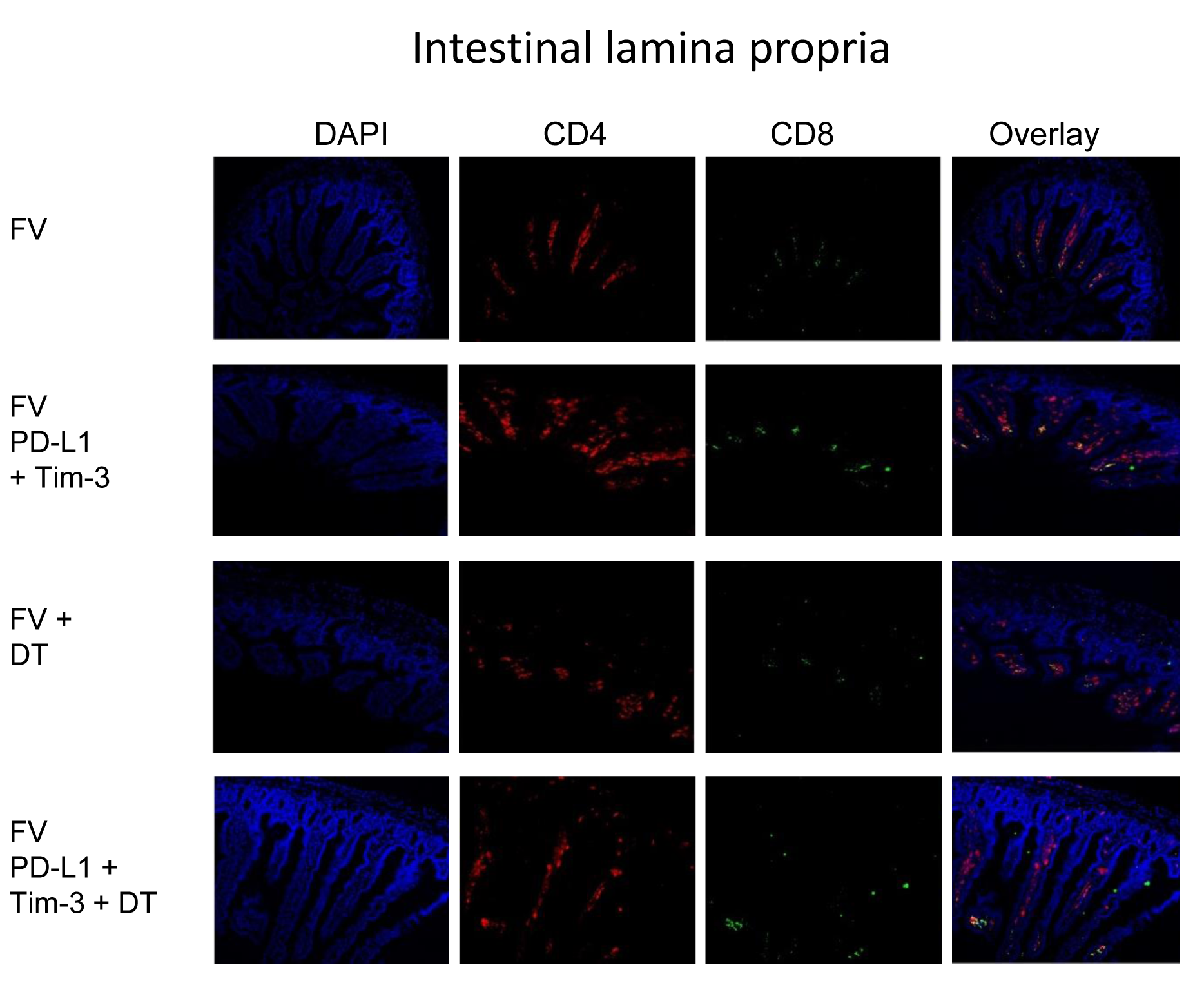

Supplement: S3 Fig — C57BL6 (groups of FV infected mice without treatment and mice with PD-L1/Tim3 treatment) and DEREG (groups with DT treatment and group of mice with combined DT and PD-L1/Tim3 treatment were infected with FV and were treated with DT and/or blocking antibodies against PD-L1 and TIM-3 as indicated during the second week of infection. The intestine sections were stained for DAPI (blue), CD4+ T cells (red), and CD8+ T cells (green). Fluorescent images were captured at 20x magnification using KeyenceBZ-9000E microscope. (TIF) [file ppat.1008340.s003.tif]

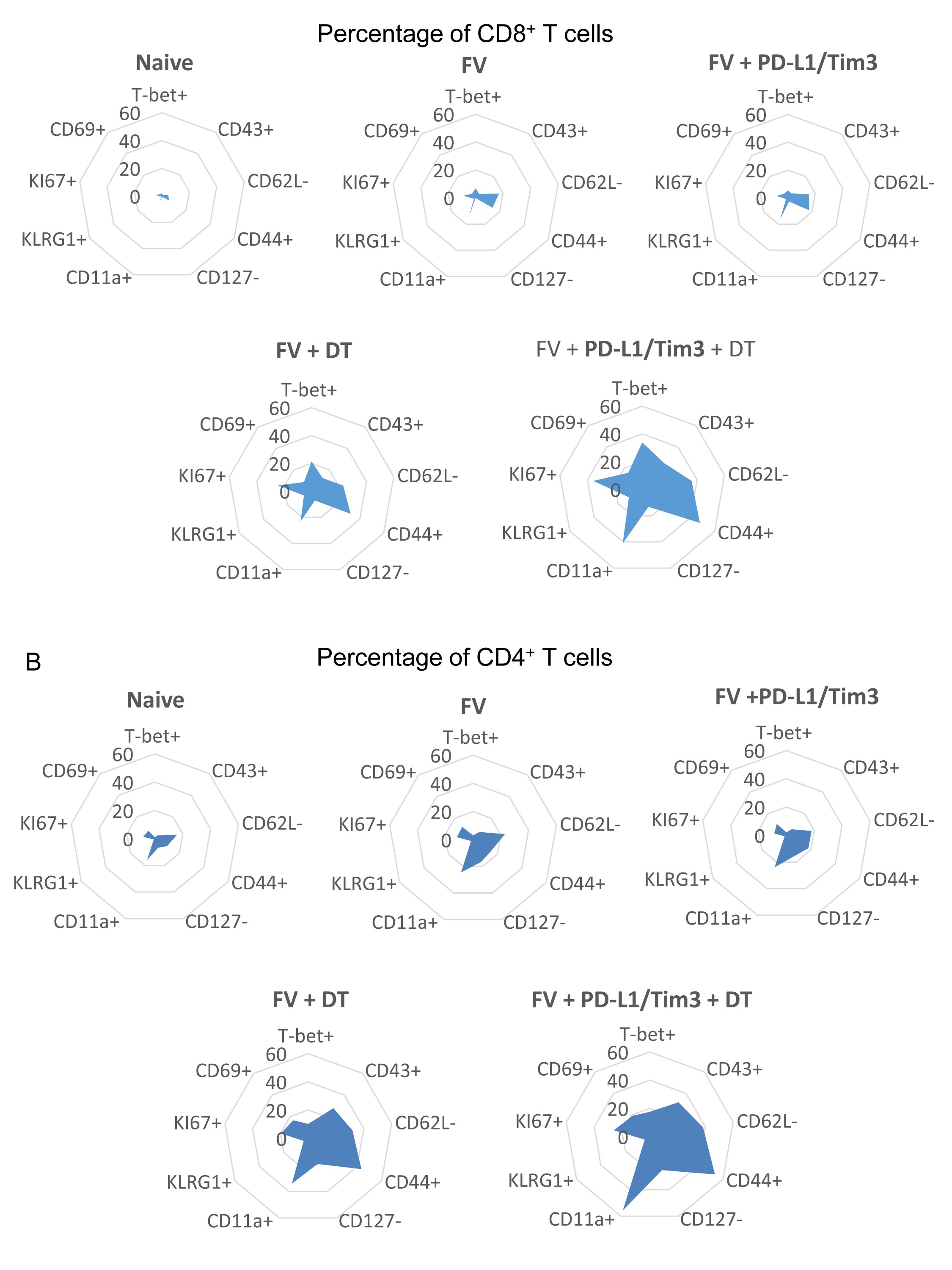

Supplement: S4 Fig — Mice were infected with FV and were treated with DT and/or blocking antibodies against PD-L1 and TIM-3 as indicated (Fig 1A). 18 days after infection mesenteric lymph nodes were isolated and the flow cytometry analysis of CD8+ and CD4+ T cells was performed. Mean percentages of CD8+ T cells (A) and CD4+ T cells (B) expressing T-bet, CD43, CD44, CD11a, KLRG1, Ki67, CD69, or negative for CD62L and for CD127 from 5–8 mice are presented. Data were pooled from 2 or 3 independent experiments with similar results. (TIF) [file ppat.1008340.s004.tif]
